# Decoding Supramolecular Packing Patterns from Computed Anisotropic Deformability Maps of Molecular Crystals

**DOI:** 10.1021/acs.jpcc.2c08212

**Published:** 2023-03-06

**Authors:** Reabetswe R. Zwane, Joaquin Klug, Sarah Guerin, Damien Thompson, Anthony M. Reilly

**Affiliations:** † School of Chemical Sciences, Dublin City University, Glasnevin, Dublin 9, Ireland; ‡ Bernal Institute, Department of Physics, University of Limerick, Limerick V94 T9PX, Ireland

## Abstract

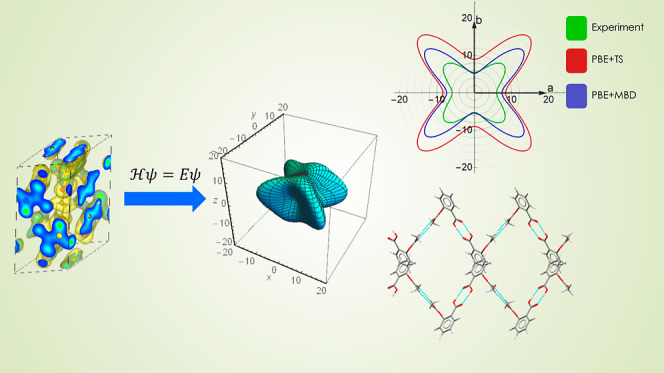

The ability to encode and embed desired mechanical properties into active pharmaceutical ingredient solid forms would significantly advance drug development. In recent years, computational methods, particularly dispersion-corrected density functional theory (DFT), have come of age, opening the possibility of reliably predicting and rationally engineering the mechanical response of molecular crystals. Here, many-body dispersion and Tkatchenko–Scheffler dispersion-corrected DFT were used to calculate the elastic constants of a series of archetypal systems, including paracetamol and aspirin polymorphs and model hydrogen-bonded urea and π–π-bound benzene crystals, establishing their structure–mechanics relations. Both methods showed semiquantitative and excellent qualitative agreement with experiment. The calculations revealed that the plane of maximal Young’s modulus generally coincides with extended H-bond or π–π networks, showing how programmable supramolecular packing dictates the mechanical behavior. In a pharmaceutical setting, these structure–mechanics relations can steer the molecular design of solid forms with improved physicochemical and compression properties.

## 1 Introduction

The crystal structure of the solid form of an organic molecule influences its stability and mechanical response, yet the rational design remains difficult, which limits opportunities for bulk mechanical properties to be engineered and tailored to specific applications.^1^ The mechanical properties dictate the key bulk properties of hardness, powder flexibility and flow, and general mechanical robustness to stress and strain during manufacturing. These properties are important for many applications of organic materials, particularly for the formulation of pharmaceuticals. Nanoindentation can provide qualitative^2,3^ and quantitative^4−6^ information on mechanical properties for a variety of materials, with only a small volume of material required to characterize properties such as hardness, Young’s modulus, and fracture toughness. However, hardness as a common measure of mechanical behavior has no fixed definition, and reported measures of hardness depend on the experimental setup and material class. The deformation process that occurs during the measurement, upon which the interpretation and results depend, is not fully understood,^7^ and more importantly, experimental conditions can significantly affect the final value.^8^ Furthermore, depending on the experimental approach, nanoindentation results may probe only specific surfaces,^6^ providing only a very limited picture of the bulk mechanical behavior. Finally, studying mechanical behavior is impossible for certain molecular crystals. For example, metastable materials are found only in extreme conditions, such as high-pressure polymorphs.

Due to these experimental challenges, there is increased interest in using computational modeling to understand and predict the mechanical response of molecular crystals. Density functional theory (DFT),^9^ particularly dispersion-corrected DFT,^10,11^ is widely applied to understanding molecular crystal structures including structure prediction^12^ and determining relative stabilities.^13^ Calculations of mechanical properties^12−14^ emphasize the importance of including dispersive van der Waals (vdW) effects, in particular many-body effects, for predictive modeling. Here, we calculate the second-order elastic constants of a broad range of organic crystals from benzene and urea to polymorphs of aspirin and paracetamol and benchmark the prediction of elastic constants for divergent classes of molecular crystals using two alternative DFT models. Benzene and urea are well-studied examples of π–π bound and hydrogen-bonded solids, respectively. Aspirin and paracetamol represent pharmaceutically relevant molecules, for which control of mechanical properties is key for improving tabletability. We trace the benchmarked mechanical properties back to key molecule sites and crystal contacts, which can guide future efforts to tailor mechanical properties by rational design.

Elastic properties are characterized by various elastic moduli, including the bulk, shear, and Young’s moduli. The moduli are expressed in terms of elastic stiffness or compliance constants, which are components of the fourth-order elastic stiffness ***C*** and compliance ***S*** tensors, respectively. Elastic stiffness constants are conventionally described within the Lagrangian strain formalism.^15^ The crystalline solid is assumed to be a homogeneous and anisotropic elastic medium. With small deformations below the fracture limit, stress and strain can be related within the linear regime using the generalized form of Hooke’s law
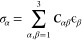
1where σ is the stress on the unit cell when strain ϵ is applied and ***C***_αβ_ is the elastic stiffness constant. The inverted form of eq 1 gives components of the compliance tensor, denoted by ***S***_αβ_. One common approach for the determination of elastic constants from the first principles is to compute the second derivative of the energy with respect to applied strain from which the second-order elastic constants ***C***_αβ_ can be extracted. Alternatively, as the stress tensor represents the first derivative of the energy, the elastic constants may be determined from its first derivative of stress with respect to strain. The symmetry of the fourth-order stiffness tensor leads to a contracted form (*via* Voigt notation), which is a symmetric 6 × 6 second-order matrix, with coefficients *C*_*ij*_.^16^ The coefficients *C*_*ij*_ are determined numerically by calculating the stress tensor as a function of varying amounts of strain generated by deforming the unit cell in specific, symmetry-dependent strain patterns.^16^

## 2 Computational Details

### 2.1DFT Calculations

The atomic coordinates of the experimentally determined crystal structures of aspirin forms I, II, and IV, paracetamol forms I and II, urea, and benzene were obtained from the Cambridge Structural Database (CSD),^17^ with the reference codes ACSALA05, ACSALA13, ACSALA23, HXACAN13, HXACAN37, UREAXX12, and BENZEN20, respectively. DFT calculations were performed for these molecular crystals using the CASTEP plane-wave periodic DFT code (v.19.11) with ultrasoft pseudopotentials.^18^ A plane-wave cutoff energy of 1000 eV was used for all calculations, and the Fourier transform grid scales for the electron density with its high-frequency components set to 2.0 and 3.0 times the wavefunction value, respectively. These values ensured the convergence of total energies to below 5 μeV/atom. The reciprocal-space Brillouin zone was sampled using a Monkhorst–Pack *k*-point grid with a spacing of at least 0.04 Å^–1^. The Perdew–Burke–Ernzerhof (PBE) exchange–correlation functional^19^ was employed for all calculations. The van der Waals (vdW) interactions were calculated by using the pairwise Tkatchenko–Scheffler (TS)^20^ and the many-body dispersion (MBD, specifically, MBD@rsSCS)^21,22^ schemes, as implemented in CASTEP.^23^ For each of the molecular crystals, full molecular and unit cell optimizations were performed using both PBE + TS and PBE + MBD, employing a finite basis set correction. For these optimizations, the convergence criteria were 5 × 10^–7^ eV/atom for the total energy, 5 × 10^–3^ eV/Å for forces, 1 × 10^–3^ Å for displacements, and 0.02 GPa for stresses. Fixed-cell optimizations were performed for elastic constant calculations (vide infra) using delocalized internal coordinates and the same total energy, force, and displacement convergence criteria.

### 2.2 Second-Order Elastic Constant Calculations

Plots of stress tensor elements *versus* strain tensor elements yield elastic constants. For the present work, the Python scripts provided with CASTEP^24,25^ were used to distort the crystal structures and analyze the resulting stress–strain plots. The maximum value of the strain used for all calculations was 0.1 GPa, with 12 points taken along each deformation pattern. For each distorted structure, the ionic positions were fully relaxed while holding the unit cell parameters fixed.

Analysis and visualization of the elastic constants and derived properties were performed using Mathematica (version 12.0)^26^ with code adapted and extended from the work of Ortiz.^27^ Analysis and visualization of the elastic constants and derived properties were performed using Mathematica (version 12.0)^26^ with the code adapted and extended from the work of Ortiz. Details of the tensor analysis of the elastic constants are outlined in Ortiz’s paper, and we now provide an example code in the Supporting Information (S1) and also state that all files are available upon request from the corresponding author.

## 3 Results and Discussion

The goal of crystal engineering is an accurate description of structure and stability, as well as the property of interest. Therefore, we begin with a brief assessment of the ability of each density functional approximation (DFA) to reproduce the experimental crystal structures after local optimization.

### 3.1 Geometries, Lattice Parameters, and Crystal Similarity Analysis

As a first test of the predictive power of the DFT models, we compare the ability of different functionals to reproduce the experimental crystal structures after optimization. Crystal packing similarity analysis^28^ is widely used in crystal structure prediction (CSP) calculations to compare experimental and computed structures, overlaying clusters of molecules from two structures, which avoids the challenges posed by different unit cell definitions and space group settings. For all the computed structures, comparing a cluster of 15 molecules between the experiment and both PBE + TS and PBE + MBD produced a valid 15/15 match. Table 1 shows the resulting rmsd_15_ values. A smaller value indicates closer agreement to the experiment, but perfect agreement is not expected due to thermal and zero-point expansion effects, which are neglected in optimized geometries, leading to an underestimation of experimental volumes and consequently deviations in rmsd values.^12^ The CSP blind tests have seen rmsd values of around 0.2–0.5 Å for DFT-optimized crystal structures, whereas force fields can exhibit larger values, particularly for flexible molecules.^12^ For the present systems and DFT methods, the rmsds are in the range of 0.01–0.31 Å, with PBE + MBD generally exhibiting lower values, except for aspirin II and paracetamol II. This indicates that both DFT methods give excellent agreement with the experimental crystal structures.

**Table 1:** rmsd_15_ Values Determined from Crystal Packing Similarity Analysis

	rmsd_15_/Å^a^	
structure (CSD ref. code, temperature)	PBE + TS	PBE + MBD
aspirin I (ACSALA05, 20 K)	0.12	0.06
aspirin II (ACSALA13, 100 K)	0.09	0.31
aspirin IV (ACSALA23, 240 K)	0.22	0.19
paracetamol I (HXACAN13, 20 K)	0.15	0.11
paracetamol II (HXACAN37, 20 K)	0.19	0.23
benzene (BENZEN20, 100 K)	0.17	0.08
urea (UREAXX12, 12 K)	0.02	0.01

a rmsd_15_ compares the PBE + TS and PBE + MBD optimized crystal structures to the corresponding experimental structure (ref. codes and temperatures are provided for each). Note that all structures gave a full 15 out of 15 matches in the packing similarity analyses.

**Figure 1:**
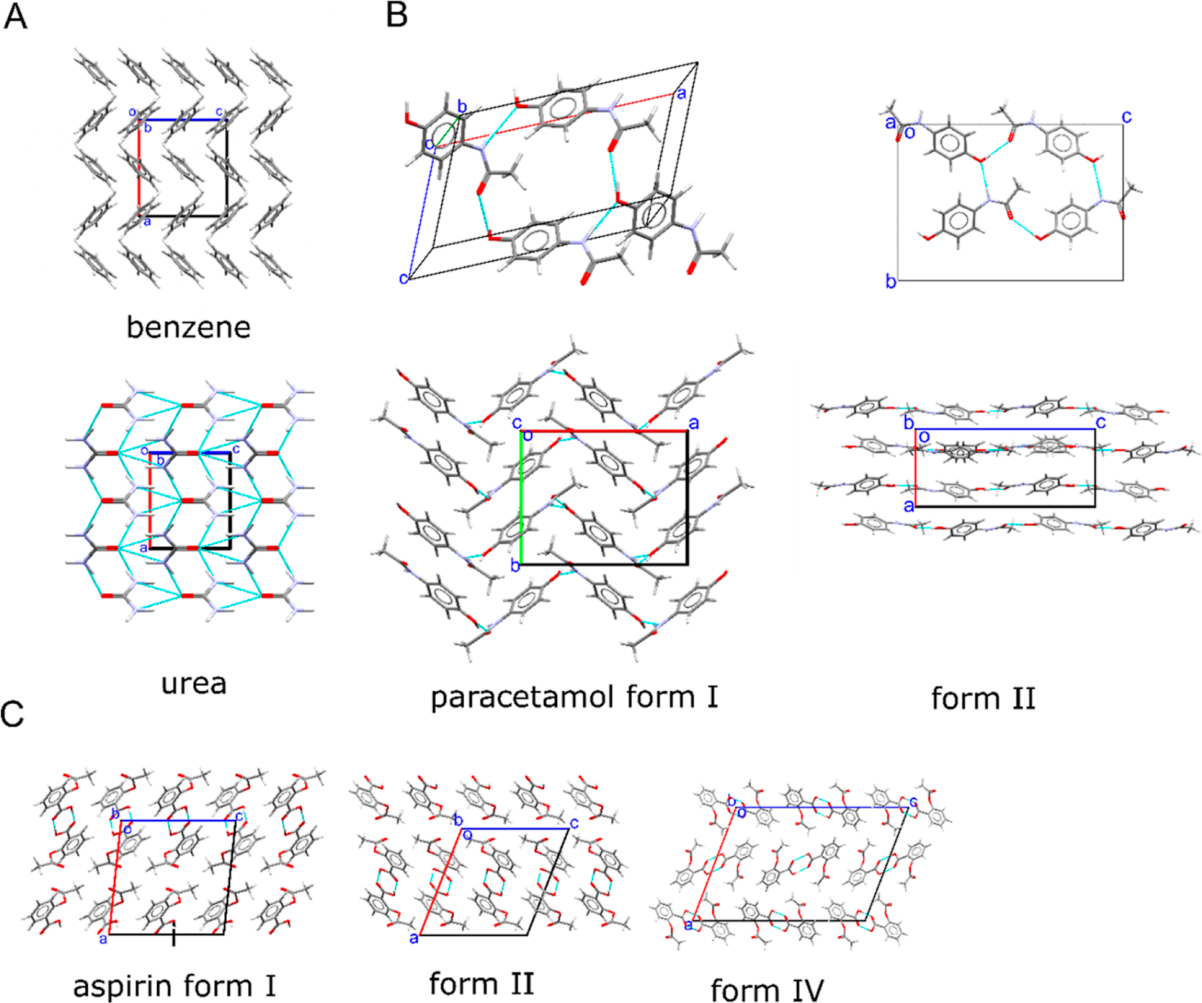
(A) Crystal packing of the orthorhombic phase of benzene and the tetragonal phase of urea, both viewed along their *b* axes, with NH···O interactions shown as thin cyan lines. (B) Crystal packing structure of paracetamol in forms I and II. The top panels show the N–H···O and O–H···O hydrogen bonds in each unit cell. The bottom panels show the crystal packing in both polymorphs viewed along the *c* axis in form I and the *b* axis in form II. (C) Crystal structures of aspirin in forms I, II, and IV, showing the crystal packing in the three polymorphs and their difference in the arrangement of layers as viewed along the *b* axis.

Table S1 in the Supporting Information gives the experimental and calculated lattice parameters of the various crystal structures under study. As expected from the small rmsd values, the agreement is generally very good with most computed lattice parameters within a few percent of experimental ones. The largest deviation is for paracetamol II, where PBE + MBD overestimates the length of the *b* axis by nearly 6%, which is not obvious from the rmsd values. The *b* axis in paracetamol II is the direction in which the paracetamol molecules form layers (see Figure 1B), and the alignment of the vdW and π–π stacking interactions in one direction and hydrogen bonding interactions in another may lead to a significant proportion of the deviations between theory and experiment being displayed in this parameter. Nevertheless, both DFT methods reproduce all the structures with good agreement.

### 3.2 Relative Stabilities of Polymorphic Forms

The second test for the assessment of DFT models is the relative stability of polymorphic forms. The predicted or modeled relative stabilities of aspirin and paracetamol have been studied^29−32^ both as useful model systems for pharmaceutical polymorphism and as demanding tests of different computational methods. Table 2 gives the total energy differences between the aspirin and paracetamol polymorphs calculated using two variants of dispersion-corrected DFT, namely, Perdew–Burke–Ernzerhof (PBE) exchange–correlation^19^ with Tkatchenko–Scheffler (TS)^20^ and the many-body dispersion (MBD)^33^ functionals, PBE + TS and PBE + MBD, respectively.

**Table 2:** DFT Total Energy Differences  per Molecule

	Δ*E*_*i*→*j*_/kJ·mol^–1^·molecule^–1^^a^	
phase change	PBE + TS	PBE + MBD
aspirin I → II	–0*.*32	0*.*68
aspirin I → IV	9*.*21	17*.*99
aspirin II → IV	9*.*54	17*.*30
paracetamol I → II	–0*.*84	–1*.*73

a The Δ*E* values are equivalent to lattice energy differences. Energies are calculated by PBE + TS and PBE + MBD for the transformation between each of the considered polymorphic forms of aspirin and paracetamol.

Forms I and II of aspirin feature very similar crystal structures^34^ as shown in Figure 1C. Both polymorphs feature centrosymmetric carboxylic acid dimers linked *via* two O–H···O hydrogen bonds and vary only in the arrangement of these dimers in layers and the resultant weak interlayer C–H···O hydrogen bonds.^4,35^ The dimers are arranged in 2-D layers parallel to the (100) plane, wherein the C–H···O contacts are the dominant interactions between the layers. The 2-D parallel layers are identical in the two polymorphs but differ in the arrangement of the layers relative to each other. Hence, the relative lattice energies for aspirin form I and form II show only a very small energy difference, in agreement with previous calculations with these DFT methods^29^ and other methods,^31,36^ suggesting that the two forms are nearly degenerate in terms of lattice energy.

In contrast to aspirin forms I and II, form IV is significantly less stable, as would be expected for a metastable form only recently identified from melt crystallization.^32^ The dimers in form IV are arranged in layers as in forms I and II, but there is a clear distinction of layer geometry in form IV as there are two symmetrically independent layers. The energy difference changes dramatically depending on the level of theory, with PBE + TS and PBE + MBD differing by over 8 kJ/mol. Previous calculations at the optB88-vdW level of theory predicted form IV to be around 8.35 kJ/mol higher in energy than forms I and II, closer to the PBE + TS energy difference.^32^ These variations suggest that form IV might be a more useful structure than forms I and II to consider for comparing the predictive power of total energy methods as energy gaps of this size and such a large difference between DFAs could see a real metastable structure omitted in CSP calculations.

For paracetamol, both levels of theory favor the stability of form II over form I, consistent with the results of Rossi *et al.*(30) In their work, they calculated the relative stabilities of paracetamol form I and form II at various levels of theory, including PBE + D3, PBE + TS, and PBE + MBD. They found the difference in lattice energy of the two polymorphic forms to be 0.29 kJ/mol^*–*1^/molecule when using PBE + D3, correctly predicting the stability of paracetamol form I over form II. In contrast, the differences per molecule when using PBE + TS and PBE + MBD were found to be −0.77 and −1.93 kJ/mol^*–*1^, respectively. However, Rossi *et al.* also performed free energy calculations based on a sophisticated treatment of vibrations which changed the ordering for these functionals to that expected from the experiment, illustrating the challenges of predicting relative stabilities.

### 3.3 Elastic Properties

Having established that the functionals closely reproduce the different crystal structures in terms of geometry and energetics, we can now analyze their elastic properties. Supporting InformationS2 includes plots of the convergence tests performed on paracetamol form I, and they show that energies, forces, and stresses are converged with respect to plane-wave cutoff energy and k-points. In addition, Table S2 in the Supporting Information presents the elastic constants for each molecular crystal calculated at the PBE + TS and PBE + MBD levels of theory. The elastic stiffness matrices of all the studied crystal structures satisfy the Born elastic stability criterion for unstressed crystals.^37^ The computation of vibrational zero-point and thermal contributions can be prohibitively expensive for full DFT on medium- to large-sized crystals, with limited studies available.^38−40^ Hence, the elastic constants and related elastic properties are determined from the 0 K calculation of the lowest temperature experimental crystal structure.^40,41^ It is important to note that the DFT elastic constants calculated at 0 K exhibit a systematic bias arising from the neglect of zero-point vibrational energy and thermal expansion. This leads to an overestimation of the elastic constants due to the underestimation of the equilibrium volume.

In addition to the standard elastic properties, such as Young’s modulus, we also consider a measure of anisotropy for each property, to quantify directional dependence. The measure of anisotropy for each modulus is defined as
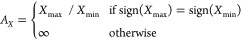
2where *X* is the elastic modulus. Calculations were performed using the open-source online code ELATE^42^ to visualize the elastic properties through the 2D and 3D spherical plots.

### 3.4 Benzene and Urea

We first considered benzene and urea as model crystals. These simple assemblies feature only a single type of intermolecular interaction, which allows us to directly relate their packing to their mechanical behavior. The stable form of benzene crystallizes in the orthorhombic space group *Pbca*. The crystal packing (Figure 1A) shows the distinctive herringbone pattern with molecules layered along the *b* axis through vdW interactions, which include π–π and C–H···π interactions.^43^ The stable form of urea crystallizes in the non-centrosymmetric tetragonal  space group and shows planar molecules arranged in a head-to-tail fashion in continuous tape motifs extending along the *c* direction (Figure 1A). The tapes are held together by a strong network of N–H···O hydrogen bonds. The adjacent tapes are orthogonal to each other and linked by secondary hydrogen bonds.

**Figure 2:**
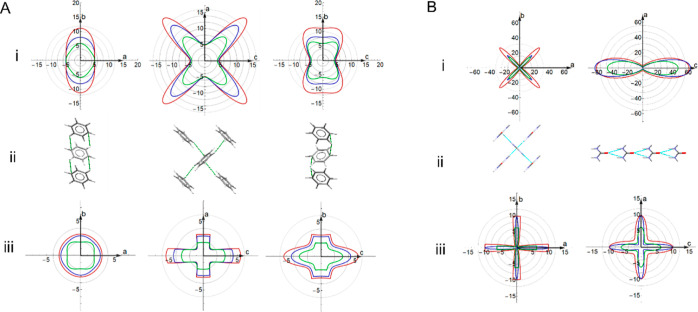
(i) 2-D spatial dependence of Young’s modulus in the *ab*, *ac*, and *bc* planes, (ii) corresponding supramolecular packing pattern, and (iii) minimum shear modulus in the *ab*, *ac,* and *bc* planes for (A) benzene and (B) urea, calculated using PBE + MBD (blue) overlaid with PBE + TS calculations (red) and experimental values^41,45^ (green). The C–H···π close contacts and hydrogen bonds in benzene and urea are shown as green and cyan dashed lines, respectively. The *ac* and *bc* planes of urea are symmetrically equivalent, and therefore, the *bc* plane is omitted.

Table 3 shows the calculated elastic properties of benzene, urea, paracetamol I, paracetamol II, aspirin I, aspirin II, and aspirin IV. For benzene, both levels of theory overestimate the absolute values of the elastic properties, reflecting the 0 K temperature. The addition of many-body vdW contributions appreciably reduces the stiffness and shear stability of the crystal, and so, PBE + MBD is in closer agreement with the experimental values. Importantly, for efficient computational screening, both levels of theory capture the correct shape of Young’s and shear moduli, which is illustrated by the 2-D spatial dependence plots in Figure 2A. For visualization and interpretation, the spatial dependence plots are spherical plots of Young’s *E*(***u***) and shear moduli *G*(***u*, *v***), where *E* is a function of the unit vector ***u*,** along which deformation occurs and *G* is additionally a function of a second vector ***v*** that is perpendicular to ***u***. During shearing, the material responds to a strain along ***u***, which is in a plane normal to ***v***. For plotting, the vectors ***u*** and ***v*** are parameterized using spherical coordinates for 3-D representations and polar coordinates for 2-D representations, where θ and φ are the parametric variables. For Young’s modulus, the resulting 3-D spherical plot is essentially a parametric surface with a radius equal to *E*(θ, φ). For the shear modulus, the parametric surface of radius *G*(θ, φ) can represent the minimum, maximum, or average shear modulus, of which the minimum is considered in this work. We invite the reader to consult the work of Nordmann for the theoretical background of the visualization of the shear modulus.^44^

**Table 3:** Voigt-Averaged Shear (*G*_V_) and Young’s Moduli (*E*_V_) (in GPa), and the Anisotropy Indices (*A*_*X*_) for the Young’s and Shear Moduli of Benzene, Urea, Paracetamol, and Aspirin, Obtained Using PBE + MBD DFT Calculations

	experiment^a^	PBE + MBD	PBE + TS	experiment^a^	PBE + MBD	PBE + TS
	Benzene			Urea		
*G*_V_ (GPa)	2.9	4.1	5.0	7.8	12.4	12.7
*E*_V_ (GPa)	7.5	10.2	12.8	20.0	30.6	32.0
*A*_G_	3.7	5.9	7.8	22.6	67.7	24.4
*A*_E_	2.4	3.4	4.0	24.4	52.9	17.7
	Paracetamol I			Paracetamol II		
*G*_V_ (GPa)		5.7	7.1		6.6	7.4
*E*_V_ (GPa)		14.8	16.9		17.4	19.3
*A*_G_		6.7	6.1		7.3	12.7
*A*_E_		4.3	4.2		7.6	14.0
	Aspirin I			Aspirin II		
*G*_V_ (GPa)	3.2	4.7	5.6		5.2	4.8
*E*_V_ (GPa)	8.4	12.4	13.1		13.7	12.8
*A*_G_	2.6	5.0	3.9		8.8	3.1
*A*_E_	2.1	3.4	3.0		6.1	3.7
	Aspirin IV					
*G*_V_ (GPa)		7.0	5.3			
*E*_V_ (GPa)		17.0	13.7			
*A*_G_		13.0	6.4			
*A*_E_		6.2	4.9			

a Experimental elastic constants for benzene measured at a temperature of 170 K,^41^ for urea at 298 K,^45^ and for aspirin I at 296 K.^35^

The DFT directionality of the two properties clearly matches qualitatively with the experiment. Young’s and shear moduli calculated with DFT are more anisotropic than those measured experimentally (Table 3). This amplification of subtle differences can, as discussed below, aid crystal design and engineering.

Comparing the 2-D plots with structural motifs extracted from the crystal structure, we can see that the elastic response aligns with the key structural features of benzene. This is particularly evident for Young’s modulus. The stiffest direction for uniaxial compression is the off-diagonal of the *ac* plane, corresponding to the diagonal T-shaped stacking of benzene molecules. These stacks are known from gas-phase calculations to be the most stable interaction between benzene dimers^46^ and therefore would likely be the hardest to compress. Young’s modulus has similar behavior in the *ab* and *bc* planes, with small difference arising from the different placement of C–H···π close contacts, which form regular ladder-shaped arrangements in the *ab* plane but the contacts alternate in the *bc* plane.

The minimum shear modulus maps directly to the intermolecular contacts. In these plots, shearing occurs in the plane tangential to the value plotted, and so, the easiest direction in which to shear is the off-diagonal in the *ac* plane that slices the T-shaped benzene dimers. In the elastic regime, this motion preserves the dimers much more than shearing in other directions. Similarly, the laddered pattern of contacts in the *ab* plane leads to more isotropic shearing than in the *bc* plane. Hence, the core structural synthon features significantly impact the elastic response.

Moving on to urea, which is dominated by hydrogen bonding, we again see good qualitative agreement between DFT and experimental maps as shown in Figure 2B. As before, DFT at 0 K overestimates the magnitudes of the ambient elastic response. As for benzene, PBE + MBD gives closer agreement with the experimental moduli but shows significantly exaggerated anisotropy (Table 1). The largest resistance of Young’s modulus is in the *c* direction, stemming from the infinite tapes of the urea structure extending in this direction and connected by strong N–H···O hydrogen bonds, as shown in Figure 2B(ii). From the *ab* plane, the cross-like arrangement of the N–H···O hydrogen bonds is reflected in the cross-like shapes of Young’s and shear moduli. Similar to benzene, where Young’s modulus is large, the shear modulus is low. Our analysis confirms that Young’s modulus is the largest in the direction that leads to maximal compression of the hydrogen bonds.

Moving to the active pharmaceutical ingredients (APIs), we first examine paracetamol. The structures of forms I (monoclinic) and II (orthorhombic) of paracetamol significantly influence their elastic properties, as has been experimentally observed and probed with calculations.^47^ The acetamide and hydroxyl groups participate in form-dependent hydrogen bond networks. In form I, the molecules pack *via* pleated or zigzag layers of hydrogen bonds with those in the adjacent layer, while form II assembles in flat layers (Figure 1B). The hydrogen bond network coupled with the interlayer steric contacts generates a more complex elastic response. The brittle crystals of form I are thought to result from the more interpenetrating hydrogen bond network, whereas form II has more compliant, more tabletable crystals,^47,48^ which has been linked to its 2-D layered vdW structure, permitting facile shear in the *ac* plane.

**Figure 3:**
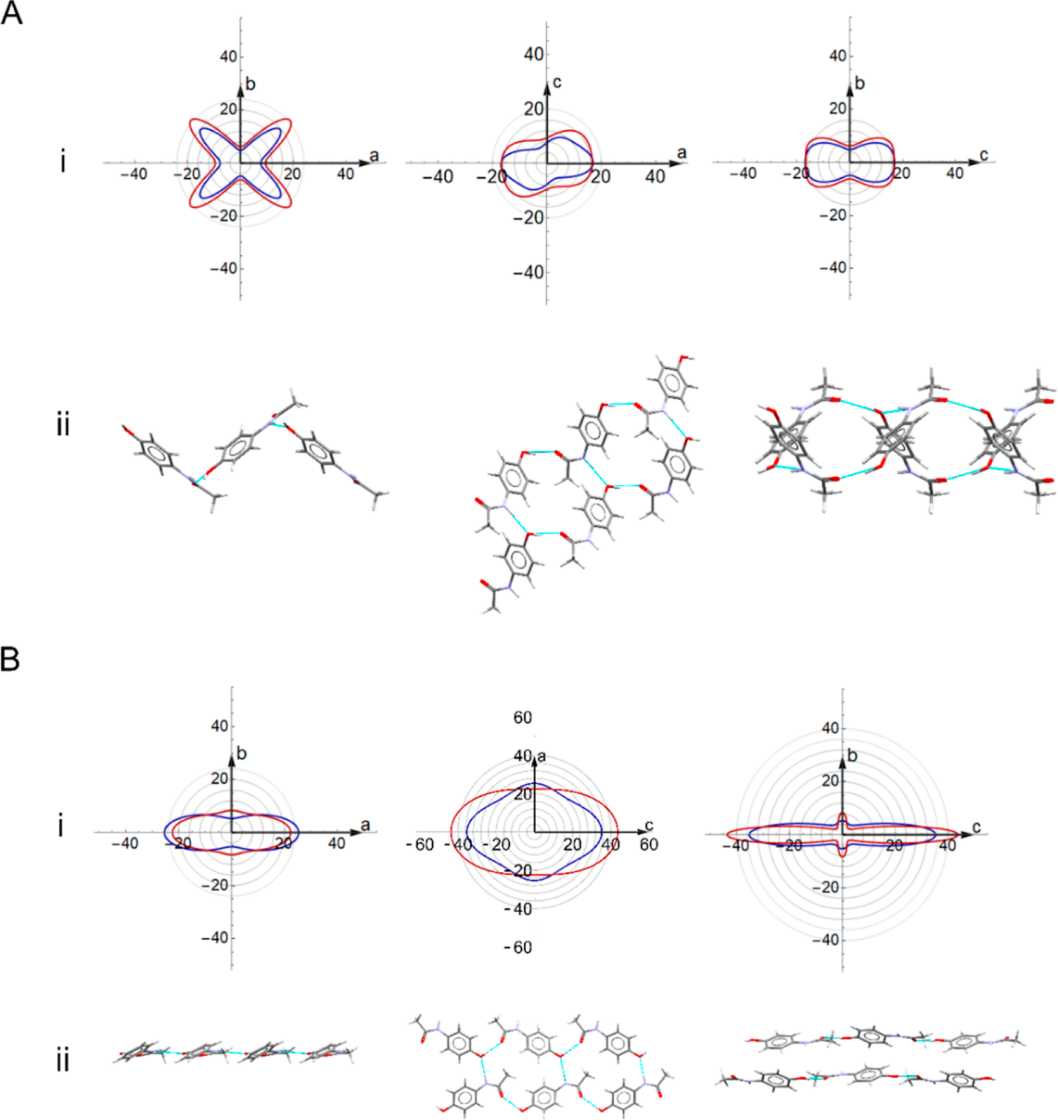
2-D spatial dependence of Young’s modulus (i) and the arrangement of the intermolecular interactions (ii) for (A) paracetamol form I and (B) form II in the *ab*, *ac,* and *bc* planes (reading left to right), calculated using PBE + MBD (blue) and PBE + TS models (red).

As for the model compounds, PBE + TS calculations on paracetamol generally show larger values of the elastic moduli than those obtained with PBE + MBD (Table 1). Both levels of theory agree qualitatively on the directionality of the elastic response, which is mapped in the 2-D spatial dependence of Young’s modulus (Figure 3). The structural differences between the two polymorphic forms are reflected in the mechanical behavior, but the relationship between the structure and the mechanical response is more complex than for the purely H-bonded urea and purely vdW-stabilized benzene. In form I, the zigzag layers produce a cross-shaped pattern of resistance to compression in the *ab* plane, corresponding to the zigzag arrangements of the N–H···O and O–H···O hydrogen bonds, as seen in Figure 3A(ii). For form I, the behavior of Young’s modulus in *ac* and *bc* is similar due to the closed-loop formation of the N–H···O and O–H···O hydrogen bonds in both planes. The closed-loop formation of the hydrogen bonds in form II leads to a marked difference in Young’s modulus. The layered structure of form II confines the N–H···O and O–H···O interactions to the *ac* plane and leads to significant anisotropy in Young’s modulus. This gives a strong difference between the *a* and *c* directions, which correspond to the directions of maximal compression. Consequently, the form II crystal strongly resists compression in the *a* and *c* directions (see Figure 3B), which in form II correspond to the directions of the N–H···O and O–H···O hydrogen bonds, respectively, while the *b* direction coincides with the interlayer vdW interactions, along which the sheets are stacked. By contrast, in form I, the N–H···O and O–H···O interactions are at an angle to the crystallographic directions and coincide with the maximal compression directions. In both cases, the hydrogen bonds increase Young’s modulus in the corresponding directions. Yet, the stability of both forms cannot solely be attributed to the strength of the hydrogen bonds according to diffraction and spectroscopic studies.^49^ Similarly, the extent of resistance seen in the mechanical behavior stems from more than just the strength of pairwise intermolecular interactions. Instead, the full picture should account for the cumulative effects that include the direction and arrangement of the supramolecular packing motifs, as accounted for by the DFT calculations.

Figure S2 in the Supporting Information shows the 2-D spatial dependence of the minimum of the shear modulus of paracetamol form I and form II. As observed for the benzene and urea test cases, a high value of Young’s modulus generally corresponds to a lower minimum shear modulus, for both paracetamol polymorphs. Compressing along a hydrogen bond network leads to large Young’s modulus, but shearing of the crystal in the same direction leaves the hydrogen bonding largely undisturbed, within the elastic limit. Therefore, shearing most easily occurs in the direction of the strong intraplanar hydrogen bonds as they remain intact.

As a final test of the ability of DFT calculations to predict the mechanical properties of API solid forms and relate those properties to the supramolecular structure, we compare the elastic behavior of three polymorphs of aspirin. Forms I and II of aspirin show a strong polymorphic relationship and similar structures^34^ (Figure 1C). Both polymorphs feature centrosymmetric carboxylic acid dimers linked *via* two O–H···O hydrogen bonds and vary in the arrangement of these dimers in layers and the resultant weak interlayer C–H···O hydrogen bonds.^4,35^ The dimers are arranged in 2-D layers parallel to the (100) plane, wherein the C–H···O contacts are the dominant interactions between the layers. The 2-D parallel layers are identical in the two polymorphs but differ in the arrangement of the layers relative to each other. Nanoindentation^4^ experiments and previous DFT calculations^29^ have shown some variation in the mechanical properties of the polymorphs, despite their similar structures. We also consider form IV, which has recently been characterized by the melt.^32^ The dimers in form IV are arranged in layers as in forms I and II, but there is a clear difference in layer geometry in form IV, which assembles in two symmetrically independent layers [Figure 1C(iii)].

**Figure 4:**
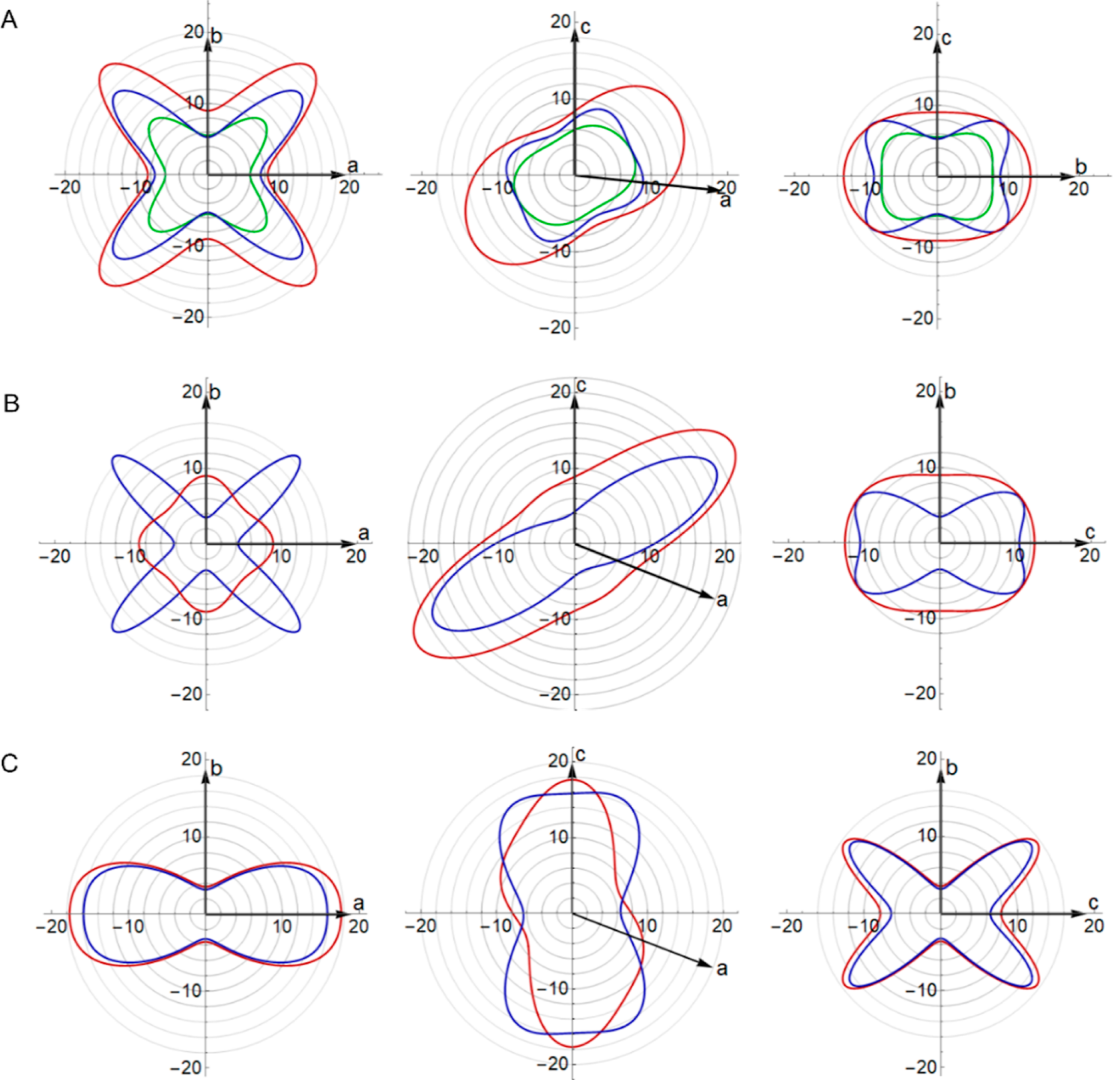
2-D spatial dependence of Young’s modulus of aspirin (A) form I, (B) form II, and (C) form IV in the ab (left), ac (middle), and bc (right) planes, calculated using PBE + MBD (blue), PBE + TS (red), and experiment (at 296 K)^49^ (green) only in the case of form I.

Figure 4 shows 2-D plots of Young’s modulus, and Figure S3 in S5 shows the 2-D plots of the shear modulus, for the aspirin polymorphs. For form I, PBE + MBD predicts Young’s modulus that is close to the experiment, with the response largely following the same trend as the experimental results (Figure 4A). For form II, there is a significant discrepancy between PBE + TS and PBE + MBD, especially in the *ab* plane, which was also observed in the previous work of Reilly and Tkatchenko.^29^ Reilly and Tkatchenko also showed that incorporating many-body dispersion has a significant effect on the calculated mechanical response of aspirin, increasing anisotropy, particularly for form II.^29^ We show in this work that all three forms show an exaggerated anisotropy as predicted by PBE + MBD compared to PBE + TS and confirmed by the larger anisotropy indices in Table 1.

**Figure 5:**
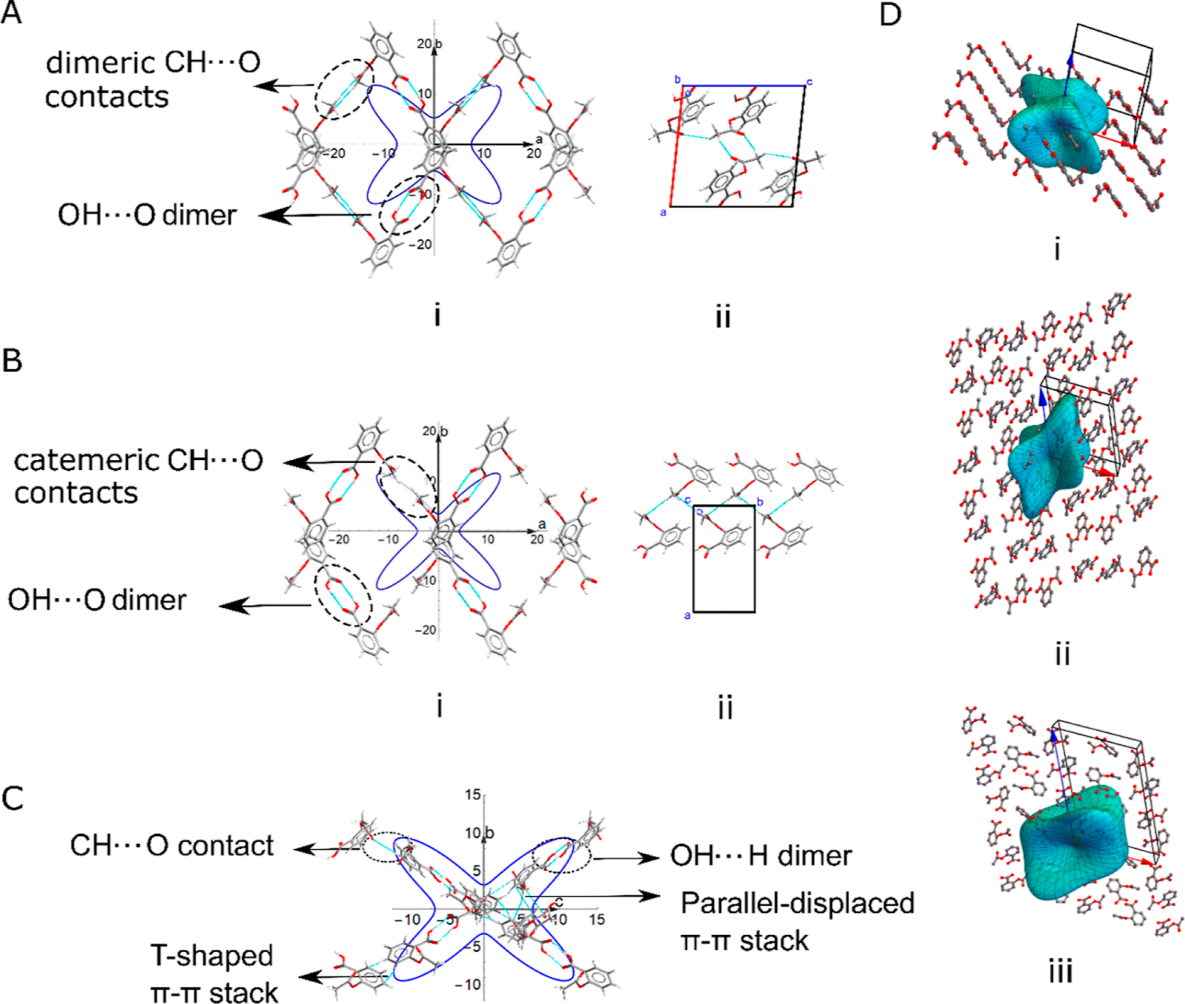
Sliced views of (i) 2-D Young’s modulus in the *ab* plane overlaid on the crystal structure, highlighting the arrangement of O–H···O hydrogen bonds and C–H···O contacts in aspirin (A) form I and (B) form II. Fragments of the crystal structure (ii) showing the (A) dimeric C–H···O interaction in form I and (B) catemeric C–H···O interactions in form II. (iii) 3-D Young’s modulus overlaid with the molecular structure and planes that lie along the dimeric C–H···O contacts in form I and along the O–H···O hydrogen bonds in form II. (C) 2-D Young’s modulus of aspirin form IV in the *bc* plane, overlaid with the arrangement of intermolecular interactions. (D) 3-D Young modulus overlaid with the molecular structure of (i) aspirin form I, (ii) form II, and (iii) form IV.

For our further analysis, we employ only the results of PBE + MBD, given its more complete treatment of dispersion^21^ and better agreement with available experimental data across our dataset. We can readily relate the directionality of Young’s modulus of the different forms to the arrangement of intermolecular contacts, in particular hydrogen bonds. Aspirin form I features O–H···O hydrogen bond dimers, as well as dimeric C–H···O interactions, between the methyl and carbonyl group connecting layers of dimers [Figure 5A(ii)]. In contrast, the C–H···O interactions in form II are catemeric [Figure 5B(ii)].^50^Figure 5A(i),B(i) shows the 2-D Young modulus and hydrogen bonding interactions in the *ab* plane for forms I and II, which contains, in both cases, the maxima of Young’s modulus. It is apparent that both the O–H···O and the dimeric C–H···O contacts in form I correspond to the direction of the highest value of Young’s modulus. Accounting for symmetry, the interactions are arranged in a butterfly-like shape, resulting in four lobes or local maxima of Young’s modulus in the *ab* plane. Similar to form I, the O–H···O hydrogen bonds and the catemeric C–H···O contacts in form II follow the same topology as the lobes of Young’s modulus in the *ab* plane.

Aspirin IV exhibits mechanical behavior similar to that of forms I and II, as seen from 2-D Young’s modulus. The different layer geometry of aspirin form IV does not significantly affect Young’s modulus. As for forms I and II, the core O–H···O dimer produces a crossed pattern in Young’s modulus, now in the *bc* plane rather than *ab*. In addition to hydrogen bonding, form IV displays π–π parallel-displaced and T-stacking arrangements (Figure 5C), further increasing Young’s modulus to 17 GPa compared to 12 and 14 GPa for form I and form II, respectively.

Since the crystal packing of aspirin presents a more intricate case compared to paracetamol, the 3-D Young moduli of aspirin form I and form II are considered for a more complete picture. Figure 5D shows 3-D Young’s moduli of the three aspirin polymorphs, overlaid on the molecular structure. All three polymorphs show four main maxima of Young’s modulus, with form II showing significant resistance in more directions compared to form I and form IV. Form II additionally shows sizable resistance in the *ac* plane [see Figures 4B and 5D(ii)], which cannot be correlated with any strong intermolecular interaction such as H-bonding as there are no contacts in that direction (see the Supporting Information, Figure S4 in S6). This suggests that the strong directional dependence of Young’s modulus is not always driven by specific pairwise interactions. The strong preference of aspirin to form dimers, as seen for all three polymorphs, leads to polymorphic forms that do not significantly differ in structure and thus show similar mechanical properties with the 3-D Young modulus of all three forms showing four main maxima. The nonvariability of the aspirin structure across polymorphic forms simplifies its properties but also limits opportunities to tune its mechanical properties by crystal engineering.

Although PBE + MBD has better agreement with the experimental values of the elastic constants, a systematic comparison between experiment and alternative models is complicated by varying degrees of error cancellation between overestimated hydrogen bonding and underestimated van der Waals effects as demonstrated in the work of Beran and colleagues.^51^ Hence, the observed exaggeration of anisotropy in the MBD maps may reflect the more realistic treatment of secondary vdW contacts. As more experimental data becomes available on the elastic properties of molecular crystals, we will be better able to compare with MBD and alternative DFT methods to more fully address this issue.

## 4 Conclusions

Computational in silico methods can help establish structure–mechanics relationships from measured crystal structures that can in turn accelerate rational engineering of pharmaceutical and other useful solid forms. For in silico predictive modeling studies, the use of an appropriate computational method is imperative for the accurate description of intermolecular interactions and thus the discovery of true structure–property relationships.

In this work, we showed that the inclusion of MBD corrections gave a significantly improved description of the elastic properties across a series of hydrogen-bonded, vdW-bonded, and more complex crystals, giving closer agreement with experiments. PBE + MBD exhibited exaggerated anisotropy of Young’s and shear moduli for most systems, particularly for the urea and the aspirin polymorphs when compared to the experiment and PBE + TS. This exaggeration may indicate the more realistic treatment of the vdW interactions by PBE + MBD.

Structure–property relationships for mechanical response were established. Intermolecular interactions, namely, hydrogen bonding and π–π stacking networks, were observed to coincide with the maxima of Young’s modulus, confirming that they confer extended strength to the mechanical behavior and thus resistance to mechanical deformation, along the corresponding crystallographic directions. An increase in the type and number of intermolecular interactions, with varying strength, complicates the structure–property relationships, as seen in increasing the complexity from the model compounds of urea and benzene, to the APIs of paracetamol, and then aspirin. The hardness of paracetamol polymorphs stems from long-range supramolecular packing patterns beyond simple pairwise interactions, showing the value of periodic DFT electronic structure calculations as a complement to pen-and-paper predictions of structure–mechanics relations. The tendency of the aspirin structure to form dimers simplifies structure prediction but restricts the engineering of significantly harder polymorphs. For crystal engineering in a pharmaceutical setting, these computationally derived structure–mechanics relationships can inform the molecular design of API formulations with improved physicochemical and compression properties.

## Supporting Information Available

Mathematical code used for the plotting of 2D and 3D representations of shear and Young’s moduli, convergence tests, experimental and calculated lattice parameters of systems considered herein, elastic constants for each molecular crystal calculated at the PBE + TS and PBE + MBD levels of theory, 2-D shear moduli plots of paracetamol and aspirin, and fragment of the aspirin form II structure in the ac plane (PDF)
