# Supplementary material for: Decoding Supramolecular Packing Patterns from Computed Anisotropic Deformability Maps of Molecular Crystals
Source: J Phys Chem C Nanomater Interfaces. 2023 Mar 6;127(11):5533–43. doi: 10.1021/acs.jpcc.2c08212 (PMC10041627; doi:10.1021/acs.jpcc.2c08212)
Supplement: Supplementary file 1 — jp2c08212_si_001.pdf [file jp2c08212_si_001.pdf]

Supplementary information

Decoding Supramolecular Packing Patterns from Computed Anisotropic Deformability Maps in Molecular Crystals

Reabetswe R. Zwane[1]; Joaquin Klug[1]; Sarah Guerin[2]; Damien Thompson[2,*]; Anthony M. Reilly[1]

* Damien.Thompson@ul.ie

1. *School of Chemical Sciences, Dublin City University, Glasnevin, Dublin 9, Ireland*
2. *Bernal Institute, Department of Physics, University of Limerick, Limerick, V94 T9PX, Ireland*

Section S1 Mathematica code

The example code supplied here as supporting material is adapted from the work of Ortiz[1] and produced using Mathematica.[2] Only the usage of the example code is provided here. We invite the reader to consult the ref. 1 for the tensorial analysis part of the code.

Examples of use of the analysis code

Determine unit cell - note definition follows crystallographic definition of a || x, b || y. We determine an angle to rotate any 3D plot around y (for monoclinic) to line up plots with any visualisation of the cell.

```
lengtha = 11.7552; lengthb = 7.13941; lengthc = 17.1714; beta = 90 Degree;
(*lattice parameters of paracetamol form I as determined by PBE+MBD*)
a = {lengtha, 0, 0}; b = {0, lengthb, 0};
c = {Cos[beta] * lengthc, 0, Sqrt[1 - Cos[beta]^2] * lengthc}; origin = {0, 0, 0}; rad = 0.1;
```

```
In[ ]:= rotangle = -1 * (beta - 90 Degree) ; (* in radians *)
```

```
In[ ]:= unitcell = Show[Graphics3D[{Red, Arrow[Tube[{origin, a}, rad]]}],
   Graphics3D[{Green, Arrow[Tube[{origin, b}, rad]]}],
   Graphics3D[{Blue, Arrow[Tube[{origin, c}, rad]]}],
   Graphics3D[{Black, Tube[{a, c + a}, rad]}], Graphics3D[{Black, Tube[{c, c + a}, rad]}],
   Graphics3D[{Black, Tube[{b, c + b}, rad]}], Graphics3D[{Black, Tube[{b + c, c}, rad]}],
   Graphics3D[{Black, Tube[{b + c, a + b + c}, rad]}],
   Graphics3D[{Black, Tube[{a + c, a + b + c}, rad]}],
   Graphics3D[{Black, Tube[{b + c, a + b + c}, rad]}], Graphics3D[
    {Black, Tube[{b, a + b}, rad]}], Graphics3D[{Black, Tube[{b + a, a + b + c}, rad]}],
   Graphics3D[{Black, Tube[{a, a + b}, rad]}], ViewProjection -> "Orthographic",
   RotationAction → "Clip", SphericalRegion -> True];
```

Set up the desired plot

```
In[ ]:= Prec = 25;
(*dataMax=Flatten[Table[{{θ,ϕ},Max[Table[ShearModulusMBD[θ,ϕ,χ],{χ,0,2 π,π/Prec}]]},
   {θ,0,π,π/Prec},{ϕ,0,2 π,π/Prec}],1];
Smaxfunc=Interpolation[dataMax];*)
dataMin =
  Flatten[Table[{{θ, ϕ}, Min[Table[ShearModulusMBD[θ, ϕ, χ], {χ, 0, 2 π, π / Prec}]]},
    {θ, 0, π, π / Prec}, {ϕ, 0, 2 π, π / Prec}], 1];
Sminfunc = Interpolation[dataMin];
```

```
In[ ]:= SminPlot = SphericalPlot3D[Sminfunc[θ, ϕ], θ, ϕ, AxesLabel → {x , y, z},
   PlotPoints → 40, Mesh → 30, LabelStyle → FontSize → 20, Mesh → 30,
   AxesStyle → Thick, ViewPoint → Front, PlotStyle → Directive[Cyan, Boxed → False],
   Axes → False, AspectRatio → Full, ViewProjection -> "Orthographic"];
```

```
In[ ]:= RotatedSminPlot = Graphics3D[GeometricTransformation[First@SminPlot,
    RotationTransform[PlotRange → {{-15, 15}, {-15, 15}, {-15, 15}}, , {0, 1, 0}]],
   Axes → False, AspectRatio → Full, ViewProjection -> "Orthographic"];
```

Load a structure from Mercury (can also be xyz format but loses bonding information; for other structures)

```
mol = Import["Path to mol2 file\\file.mol2", "MOL2"][[1]];
opa = 1.0;
molplot =
 MoleculePlot3D[mol, ColorRules → {"N" → Opacity[opa, Blue], "O" → Opacity[opa, Red],
    "C" → Opacity[opa, Gray], "H" → Opacity[opa, LightGray]}, PlotTheme → "HeavyAtom"];
```

In[ ]:=
```
scaling = 3.5; (* scale the Young's modulus relative to the crystal structure*)
surfaceopacity = 0.3;
YModPlot = SphericalPlot3D[YoungModulusMBD[θ, ϕ] / scaling, θ, ϕ,
   PlotRange → {{-15, 15}, {-15, 15}, {-15, 15}}, AxesLabel → {x , y, z},
   LabelStyle → FontSize → 20, PlotPoints → 40, Mesh → 30, AxesStyle → Thick,
   PlotStyle → Directive[Cyan, Boxed → False, Opacity[surfaceopacity]],
   Axes → True, AspectRatio → 1, ViewProjection -> "Orthographic",
   RotationAction → "Clip", SphericalRegion -> True, Boxed → False];
RotatedYModPlot = Graphics3D[GeometricTransformation[
    First@YModPlot, RotationTransform[rotangle, {0, 1, 0}]]];
Show[RotatedYModPlot, unitcell, molplot, Graphics3D[{Opacity[surfaceopacity]}],
 ViewProjection -> "Orthographic", RotationAction → "Clip",
 SphericalRegion -> True, Boxed → False, AspectRatio → 1]
```

Out[ ]=

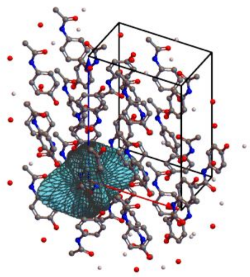

Test code for axes on polar plots (note b || y and c || z (not crystallographic convention))

```
In[ ]:= axeslength = 50;
YModxzMBD = PolarPlot[YoungModulusMBD[θ, π], {θ, 0, 2 π},
   PlotRange → {{-axeslength - 2, axeslength + 2}, {-axeslength - 2, axeslength + 2}},
   PlotStyle → {Thick, Darker[Blue]},
   PolarGridLines → {None, {4, 8, 12, 16, 20, 24, 28, 32, 36, 40}},
   Frame → False, FrameStyle → Thick, FrameLabel →
    {"\!\(\*StyleBox[\"y\",FontSlant->\"Italic\"]\) / GPa",
     "\!\(\*StyleBox[\"z\",FontSlant->\"Italic\"]\) / GPa"},
   TicksStyle → {FontSize → 24}, LabelStyle → {FontSize → 24, Black}];
(* Plot arrows for the crystal axes based on the maximum Young's modulus. Note
 that the plot is strictly 2D so does not know which axis is which,
so you must figure this out yourself and adjust accordingly. Manually
 adds cell axes on plot for viewing along side crystal structure. *)
hortaxis = Graphics[{Thickness[0.007],
    Arrowheads[0.05], Black, Arrow[{{0, 0}, {axeslength, 0}}]}];
vertlabel = Graphics[{FontSize → 24, Text["c", {4, 30}]}];
vertaxis =
  Graphics[{Thickness[0.007], Arrowheads[0.05], Black, Arrow[{{0, 0}, {0, 30}}]}];
hortlabel = Graphics[{FontSize → 24, Text["a", {axeslength, 4}]}];
Show[YModxzMBD, YModxzTS, vertaxis, hortaxis, vertlabel, hortlabel]
```

Out[ ]=

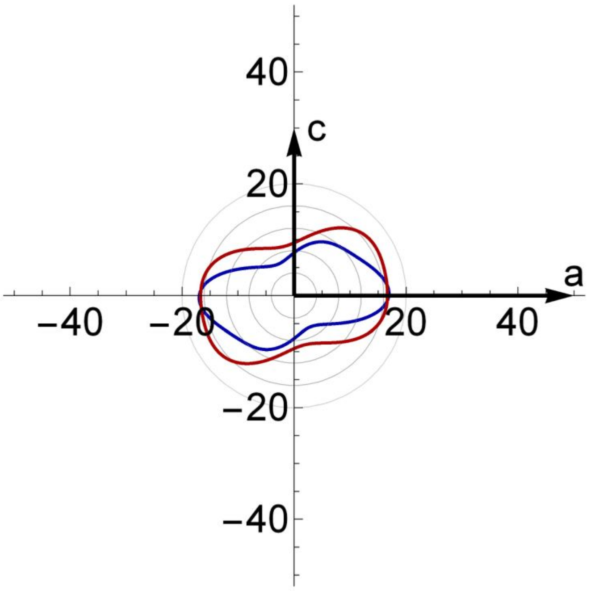

All files, including simulation input and output files and analysis scripts, are available upon request from the corresponding author.

Section S2 Convergence Tests

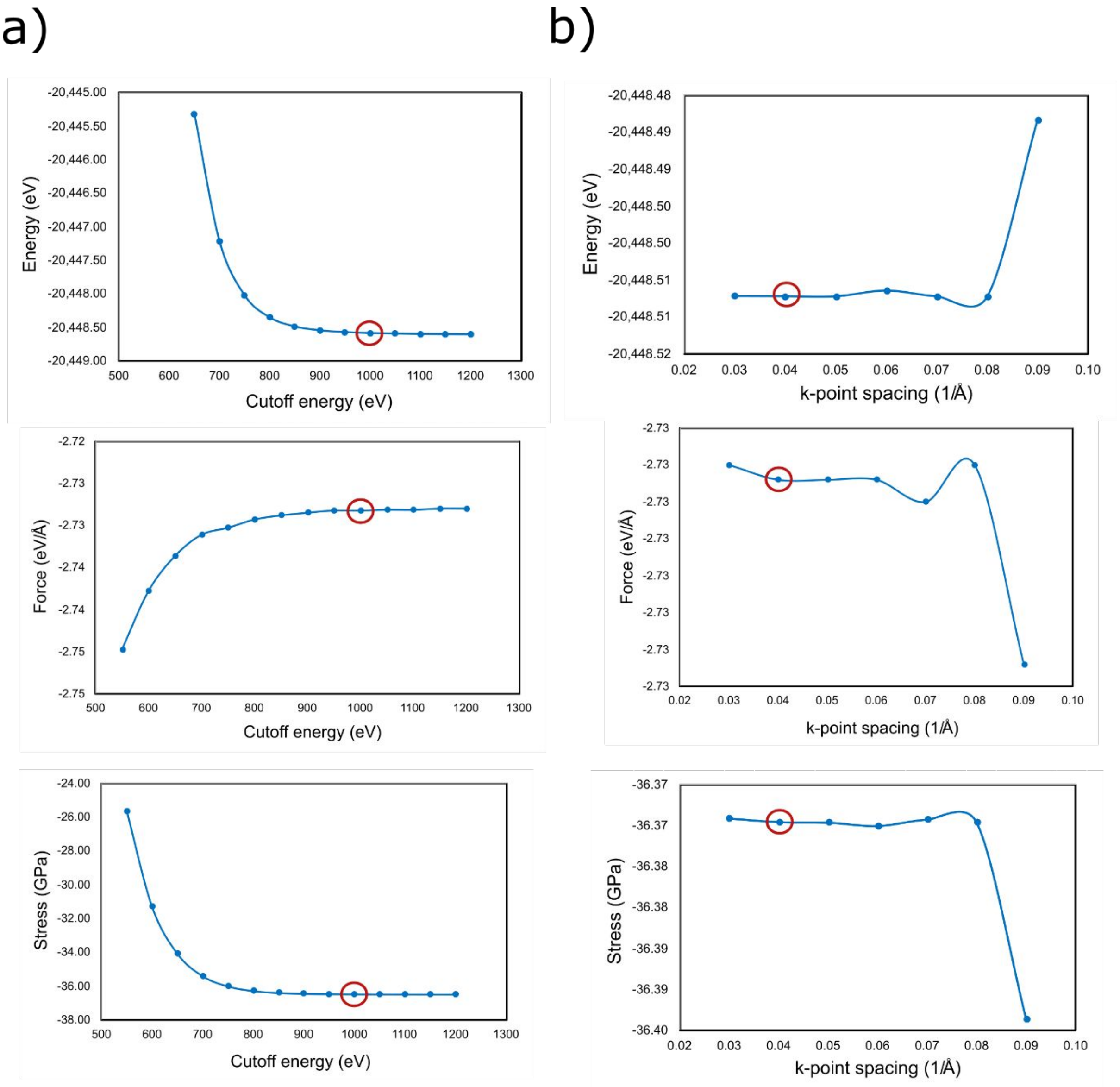

Figure S1 Computed energy, force and stress *vs.* (a) cutoff energy and (b) k-point spacing equilibration curves for paracetamol form I showing the suitability of the cutoff energy of 1000 eV and k-point spacing of 0.04 /Å.

Section S3 Lattice Parameters

Table S1: Calculated lattice parameters optimized using PBE-TS and PBE-MBD (experimental values from The Cambridge Structural Database[1])

Method, CSD ID	Space group	a/Å	b/Å	c/Å	α/°	β/°	γ/°	Volume/Å^3
				Benzene				
Expt. (100 K), BENZEN20	*Pbca*	6.78	7.41	9.45	90.00	90.00	90.00	474.99
DFT-TS	*Pbca*	6.83	7.34	9.19	90.00	90.00	90.00	460.80
DFT-MBD	*Pbca*	6.69	7.32	9.53	90.00	90.00	90.00	467.03
				Urea				
Expt. (12 K), UREAXX12	$P42_1m$	5.57	5.57	4.68	90.00	90.00	90.00	145.06
DFT-TS	$P42_1m$	5.56	5.56	4.69	90.00	90.00	90.00	144.93
DFT-MBD	$P42_1m$	5.55	5.55	4.67	90.00	90.00	90.00	144.13
				aspirin I				
Expt. (20 K) ACSALA05	$P2_1$/c	11.19	6.54	11.22	90.00	96.07	90.00	816.00
DFT-TS	$P2_1$/c	11.28	6.50	11.33	90.00	97.25	90.00	824.15
DFT-MBD	$P2_1$/c	11.24	6.51	11.31	90.00	96.03	90.00	823.81
				aspirin II				
Expt. (100 K), ACSALA13	$P2_1$/c	12.10	6.49	11.32	90.00	111.51	90.00	829.05
DFT-TS	$P2_1$/c	11.97	6.45	11.39	90.00	110.56	90.00	824.18
DFT-MBD	$P2_1$/c	12.55	6.15	11.40	90.00	111.07	90.00	820.68
				aspirin IV				
Expt. (240 K), ACSALA23	$P2_1$/c	16.74	4.79	23.80	90.00	111.08	90.00	1782.81
DFT-TS	$P2_1$/c	16.73	4.56	23.83	90.00	109.96	90.00	1707.04
DFT-MBD	$P2_1$/c	16.56	4.74	23.46	90.00	110.39	90.00	1728.07
				paracetamol I				
Expt. (20 K), HXACAN13	$P2_1$/n	7.07	9.17	12.67	90.00	115.51	90.00	741.16
DFT-TS	$P2_1$/n	7.01	9.03	11.74	90.00	99.16	90.00	733.39
DFT-MBD	$P2_1$/n	7.02	9.14	11.69	90.00	98.58	90.00	741.54
				paracetamol II				
Expt. (20 K),	*Pbca*	11.76	7.14	17.17	90.00	90.00	90.00	1441.11

HXACAN37								
DFT-TS	*Pbca*	11.63	7.16	17.24	90.00	90.00	90.00	1435.46
DFT-MBD	*Pbca*	11.45	7.55	17.19	90.00	90.00	90.00	1487.24

Section S4 Elastic Constants

Table S2: Calculated non-zero elastic constants c_{ij} in GPa for each molecular crystal, Voigt-averaged bulk and shear and Young's moduli. Levels of theory used are PBE+TS and PBE+MBD.

	benzene		urea		aspirin						paracetamol			
	Form I		Form I		Form I		Form II		Form IV		Form I		Form II	
	DFT-TS	DFT-MBD	DFT-TS	DFT-MBD	DFT-TS	DFT-MBD	DFT-TS	DFT-MBD	DFT-TS	DFT-MBD	DFT-TS	DFT-MBD	DFT-TS	DFT-MBD
c_{11}	12.23	10.57	19.60	15.91	18.31	16.34	18.91	16.33	22.18	20.03	23.40	17.78	29.96	35.11
c_{22}	15.43	10.40			19.91	14.24	19.28	16.16	11.44	10.14	16.80	12.78	10.02	7.37
c_{33}	12.22	9.51	69.74	69.82	17.65	14.80	20.18	19.63	22.51	17.87	24.50	23.06	53.34	43.35
c_{44}	5.44	4.35	9.83	9.80	4.74	5.09	5.29	5.11	5.49	5.69	7.07	5.50	0.87	1.64
c_{55}	10.74	8.28			5.05	3.59	7.05	6.62	5.46	16.03	6.97	4.22	11.08	8.98
c_{66}	3.12	2.88	22.18	20.47	9.22	8.87	2.14	7.02	4.43	4.37	12.81	10.30	3.50	3.50
c_{12}	6.30	4.38	17.78	15.30	13.15	10.95	13.01	13.54	6.51	6.26	14.99	10.94	6.66	7.79
c_{13}	9.43	7.19	12.82	10.56	8.30	7.35	9.35	8.31	4.47	5.16	12.02	9.07	16.52	14.55
c_{15}					-1.17	-1.48	1.77	0.99	-0.54	-0.31	0.02	-0.07		
c_{23}	7.03	4.51			8.15	9.16	7.89	8.58	12.52	10.68	11.14	8.59	5.98	14.55
c_{25}					1.39	-0.22	-1.09	-1.35	0.04	0.48	1.75	1.77		
c_{35}					-1.29	0.16	4.79	4.67	1.88	1.41	0.79	0.64		
c_{46}					0.54	0.06	0.93	-0.28	1.21	1.17	1.48	1.68		
Bulk modulus	9.49	6.96	21.75	19.39	12.79	11.14	13.20	12.55	11.46	10.25	15.67	12.31	16.85	15.98
Shear modulus	5.00	4.06	12.74	12.36	5.55	4.70	4.77	5.20	5.25	6.95	7.14	5.67	7.37	6.61
Young's modulus	4.56	4.35	31.97	62.68	13.10	8.86	12.55	10.52	7.85	17.00	16.69	16.92	43.61	35.24

Section S5 Shear modulus

Paracetamol

Figure S2 shows the 2D spatial dependence of the minimum of the shear modulus. Again, it is apparent that in all three planes, form II has a smaller or similar minimum shear modulus in all directions compared to form I, which is consistent with its easier tabletability.[3] The smaller minimum shear modulus of form II is also consistent with the smaller values of the shear components, C_{44}, C_{55} and C_{66}, of form II compared to form I.

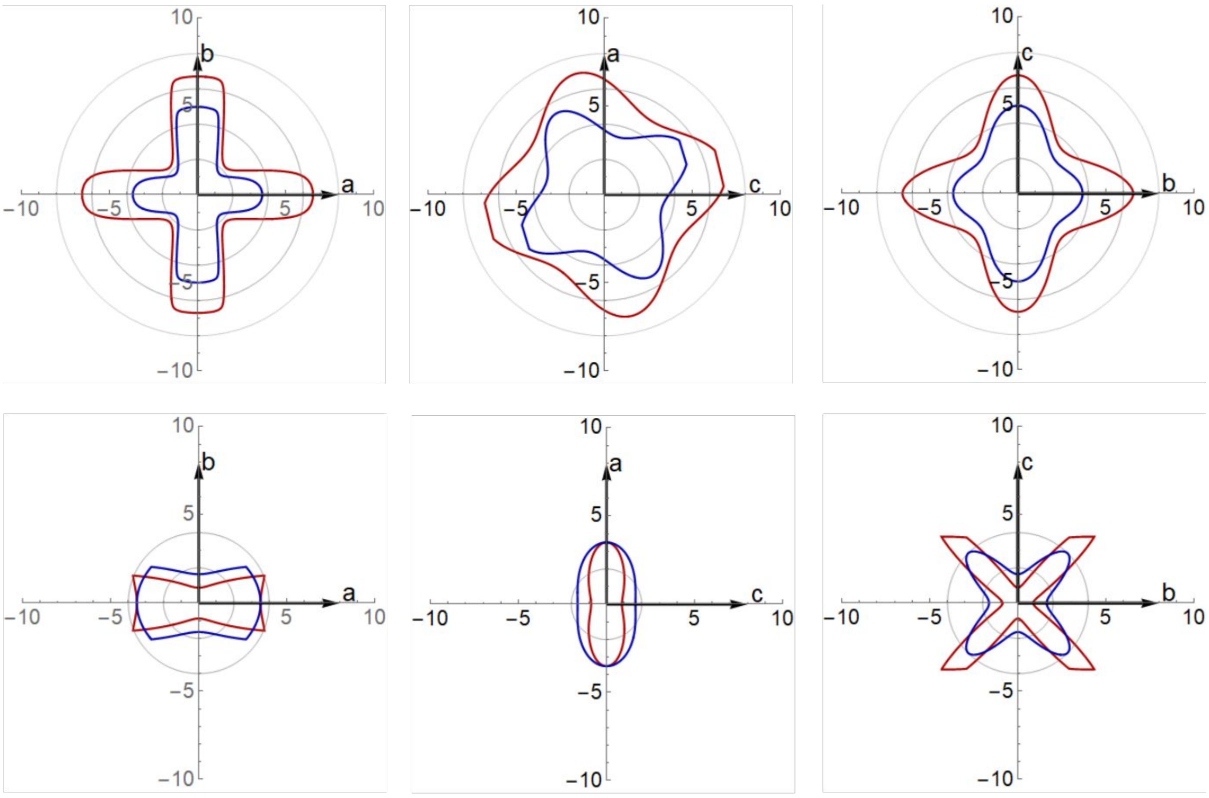

a) b) c)

Figure S2 2–D spatial dependence of the minimum shear modulus for paracetamol form I on the top panel and form II on the bottom panel in the plane (a) *ab*, (b) *ac*, and (c) *bc*, calculated using PBE+TS (red) and PBE+MBD (blue).

Aspirin

As with the Young's modulus, the shear modulus of aspirin form II is not well reproduced by PBE+TS. Furthermore, PBE+TS predicts considerably different shearing behaviour between form I and form II. Conversely, PBE+MBD predicts shearing behaviour that is similar to experiment[4] (Figure S3) in the case of form I while capturing the similarity of the mechanical behaviour between the two polymorphs.

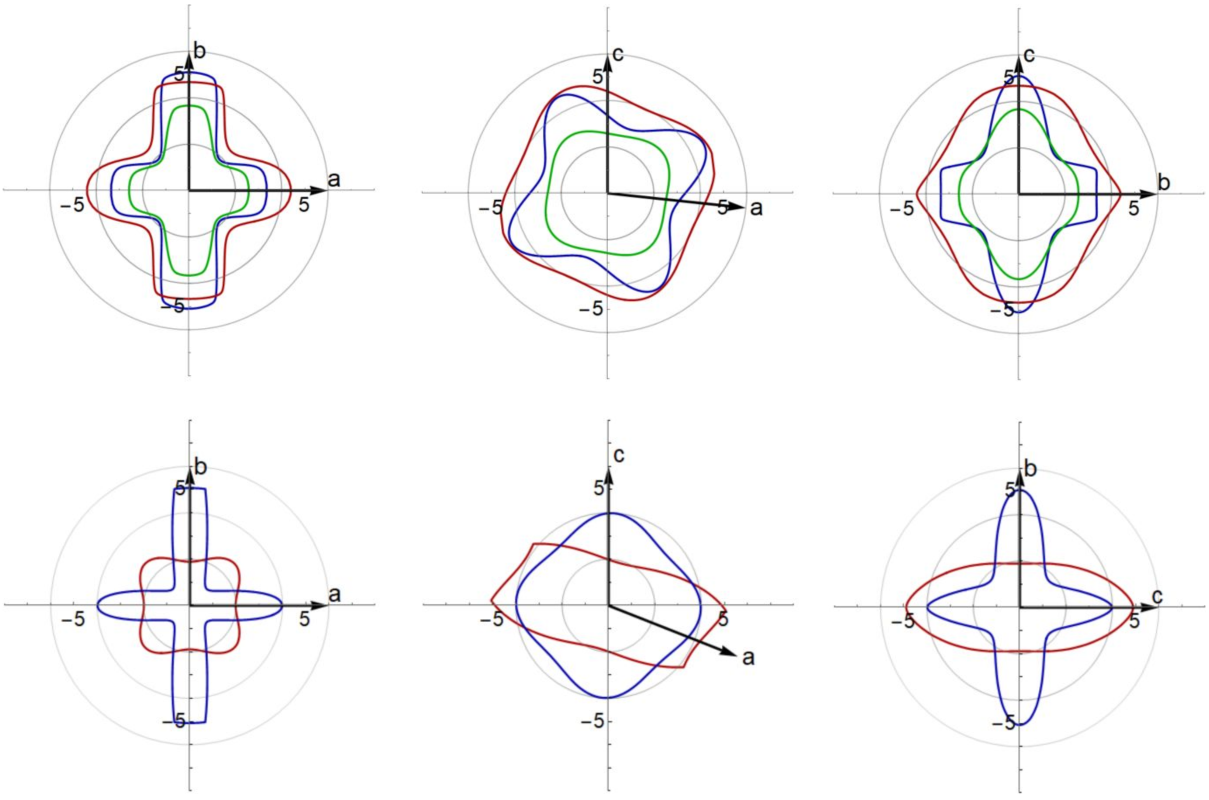

a) b) c)

Figure S3 2–D spatial dependence of the minimum shear modulus of aspirin form I (top panel) and form II (bottom panel) in the plane (a) *ab*, (b) *ac* and (c) *ab*, calculated using PBE+TS (red), PBE+MBD (blue) and determined by experiment (green).

Section S6 Fragment of aspirin form II in the *ac* plane

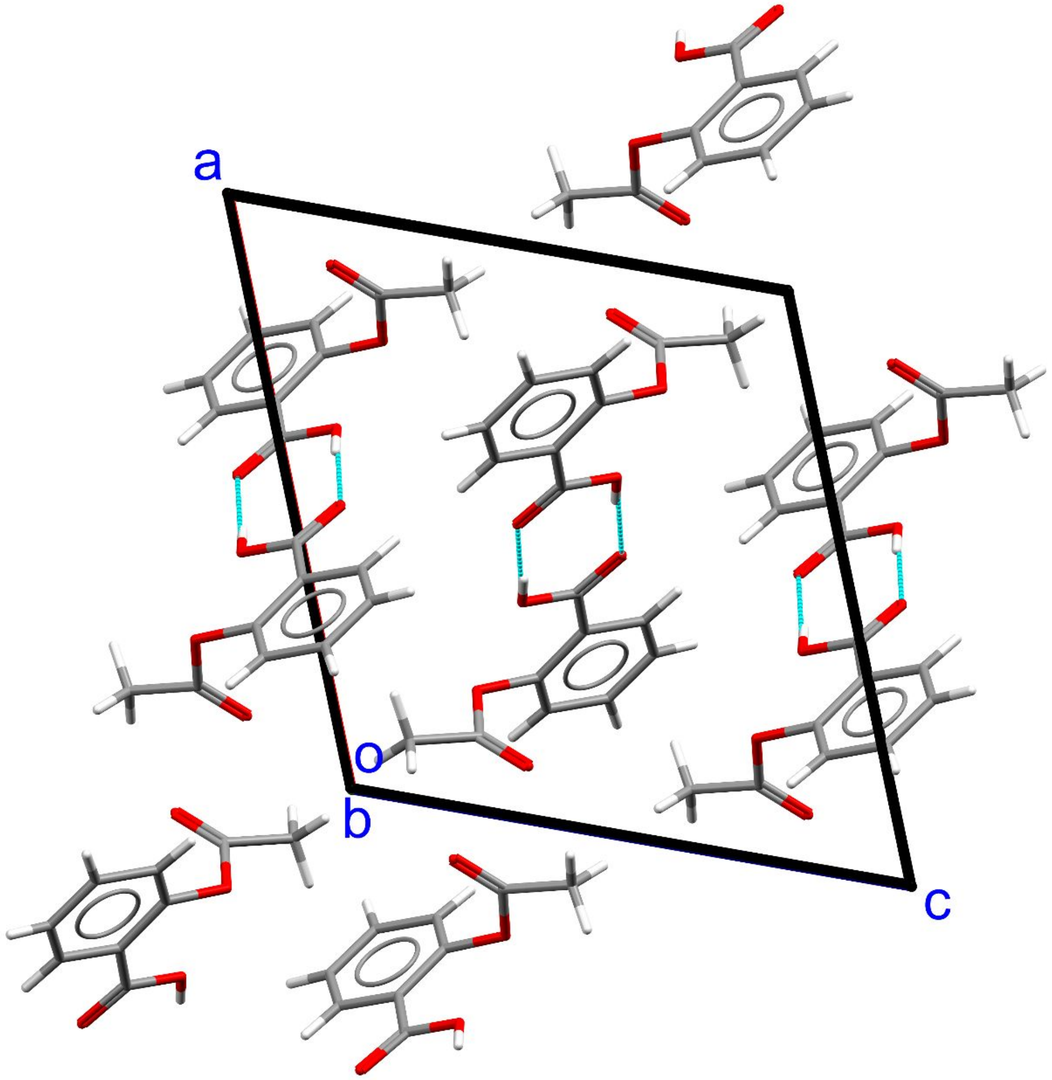

Figure S4 Fragment of the aspirin form II structure in the *ac* plane showing there are no strong intermolecular interactions in this plane.

SUPPLEMENTARY REFERENCES

1 A. U. Ortiz, A. Boutin, A. H. Fuchs and F. X. Coudert, Metal-organic frameworks with wine-rack motif: What determines their flexibility and elastic properties?, *Journal of Chemical Physics*, 2013, **138**, 174703.

2 I. Wolfram Research, Wolfram Research, Inc., Champaign, Illinois, 2022.

3 E. Joiris, P. di Martino, C. Berneron, A. M. Guyot-Hermann and J. C. Guyot, Compression behavior of orthorhombic paracetamol, *Pharm Res*, 1998, **15**, 1122–1130.

4 S. Varughese, M. S. R. N. Kiran, K. A. Solanko, A. D. Bond, U. Ramamurty and G. R. Desiraju, Interaction anisotropy and shear instability of aspirin polymorphs established by nanoindentation, *Chem Sci*, 2011, **2**, 2236–2242.
